# COVID-19 Vaccination during Pregnancy and Lactation: Attitudes and Uptakes before and after Official Recommendations in Germany

**DOI:** 10.3390/vaccines11030627

**Published:** 2023-03-10

**Authors:** Carsten Hagenbeck, Janine Zöllkau, Martina Helbig, Tanja Fehm, Nora K. Schaal

**Affiliations:** 1 Department of Obstetrics and Gynecology, University Hospital Düsseldorf, 40225 Düsseldorf, Germany; 2 Department of Obstetrics, University Hospital Jena, 07747 Jena, Germany; 3 Department of Experimental Psychology, Heinrich-Heine-University Düsseldorf, 40225 Düsseldorf, Germany

**Keywords:** COVID-19 vaccine, vaccine acceptance, pregnancy, lactation, breastfeeding

## Abstract

Background: Vaccination against COVID-19 is an effective measure to mitigate the pandemic. Pregnant and breastfeeding women were not included in registration studies, so official recommendations to vaccinate this vulnerable group appeared belated. Therefore, our aims were to evaluate vaccination uptake, reasons for and against vaccination, and the changes in these depending on the official national recommendations in Germany. Methods: An anonymous online cross-sectional survey among pregnant and breastfeeding women was conducted prior to and after the publication of the official vaccination recommendation. Results: Data from the convenience sample of 5411 participants (42.9% pregnant; 57% breastfeeding) were analysed. The recommendation was known to 95% of the participants. The information was obtained mainly autonomously (61.6%) and through the media (56.9%). Vaccination uptake increased in pregnant (2.4% before vs. 58.7% after) and breastfeeding women (13.7% vs. 74.7%). As reasons to get vaccinated, pregnant women indicated more fear of the infection than of the side effects of vaccination (52.0% before vs. 66.2% after), intended protection of the baby and oneself (36.0% vs. 62.9%), and limited information about vaccination (53.5% vs. 24.4%). Conclusion: The official national recommendation is widely known and mostly obtained autonomously, thereby showing a high level of awareness and a rise in vaccination uptake. Nonetheless, targeted education campaigns focusing on scientific evidence should be maintained, whereas the engagement of health professionals should be enhanced.

## 1. Introduction

The COVID-19 pandemic is one of the great challenges of our time, along with climate change, the depletion of natural resources, and wars. It has been shown that young women of childbearing age, in particular, suffer from the effects of the pandemic [1]. Vaccination against COVID-19 is an important intervention to combat the pandemic [2]. It is therefore important to understand the factors that cause vaccination hesitancy in order to use them as a useful basis for implementing strategic intervention instruments. The aim of these interventions is to improve vaccination adherence, with particular awareness of special population groups such as pregnant women. It is well known that trust in the health system is an important factor in the implementation of official vaccination recommendations [3]. However, evidence is scarce for the obstetric target group on the extent of implementation of official COVID-19 recommendations in the population. Therefore, we aim to close this gap with our study. In order to counsel pregnant and breastfeeding women—as well as to provide necessary information and scientific data—it is essential to analyse the reasons for and against vaccination, as well as the influence on and changes in attitudes before and after the announcement of the official vaccination recommendation.

The principal background is the fact that pregnant and breastfeeding women were not included in registration studies, such that official recommendations to vaccinate this vulnerable group appeared belatedly [4,5]. Thus, in the first half of 2021, only German gynaecological and obstetric societies recommended vaccination for pregnant and breastfeeding women [6]. As recently as September 2021, the German Standing Vaccination Committee (STIKO) at the Robert Koch Institute explicitly included pregnant and unvaccinated breastfeeding women as target groups to be vaccinated in the official COVID-19 vaccination recommendation: “[…] pregnant (from the 2nd trimester) and breastfeeding women be vaccinated with two doses of an mRNA vaccine […]” [7]. How and to what extent this change in recommendation reached the obstetric target group has not yet been explored. Moreover, it is unclear how this official recommendation was enforced and what effect it had on vaccination behaviour.

In many countries, such as Germany, a national immunisation register does not exist, especially with regard to the obstetric target groups. The estimated vaccination coverage rate in the overall population in Germany increased rapidly from 12% in April to 71% in November [8,9]. Therefore, representative survey data is required to better reflect the prevalence of vaccination and to identify gaps in the vaccination uptake among pregnant and lactating women. Since there is evidence that pregnant women are at increased risk of severe disease from COVID-19 compared to non-pregnant women [10].

Consequently, the aim of this study was to investigate the impact of the official recommendations on vaccination in the obstetric target group of pregnant and breastfeeding women during the pandemic.

The study objectives were: (a) to assess the knowledge of pregnant and breastfeeding women about the official national vaccination recommendations; (b) to identify the sources of information used; (c) to assess the vaccination coverage, as no national register exists; (d) to identify the reasons for and against vaccination before and after the official recommendations and their dynamics.

## 2. Methods

An anonymous, online, cross-sectional survey was conducted between 30 March and 19 April 2021 and between 5 and 22 November 2021. For comprehensibility, the two time intervals are subsequently referred to as before and after the official recommendation. All pregnant and breastfeeding women in Germany were eligible and formed the target population. Thus, using the Raosoft sample size calculator, a sample of at least 384 individuals for each group was required to study the selected variables (pregnant *n* = 733,144 in 2020 and breastfeeding women estimated to be as many as pregnant), assuming a response rate of 50%, a confidence level of 95%, and a margin of error of 5%.

Participants were recruited via the professional and private networks of midwives and gynaecologists and via social media (e.g., Facebook, Instagram). The survey was administered through the platform soscisurvey.de [11]. Participants provided informed written consent at the beginning of the online survey. The time for completion was approximately 10 min.

### 2.1. Materials and Procedure

A questionnaire was developed for the survey that was conducted before the official recommendation. This survey was designed to help assess the COVID-19 vaccination uptake among pregnant and breastfeeding women, as well as the reasons for and against vaccination. With a different study focus that is not part of and does not interfere with the present analysis, data from the first survey were published independently and prior to the release of the official recommendation [12].

For the purposes of the second survey after the official recommendation, the questionnaire was slightly adjusted to fit the current situation of vaccine possibilities, and a question was added regarding the level of awareness and how knowledge of the official recommendation was obtained. The following questions were dropped after the first survey because they were no longer appropriate: willingness to be vaccinated if they would receive an offer now; preferable vaccines. In order to identify general vaccination opponents from those who were not vaccinated solely because of pregnancy or lactation, we asked an additional question on these issues in the second questionnaire. Additionally, demographics and pre-existing health issues were queried. Comparisons were made between the survey results.

A total of 6128 women started the questionnaire, of whom 88.3% finished the survey. Only complete surveys were included in the analysis.

### 2.2. Measures

Participants were asked if they were pregnant or breastfeeding, which served as a filter for the following specific questions for each group: pregnancy details (i.e., gestational age, risk pregnancy (yes/no); parity (primi/multi) for the pregnancy group; and age of the child (for the breastfeeding group) were administered. Then, the following topics for both groups were evaluated: pre-existing vaccinations against COVID-19, as well as reasons for and against vaccination (multiple-select questions). For the questions that asked about the reasons for and against vaccination, participants were able to select several reasons that impacted their decision. Single-select questions were used to ask about vaccination refusal. The answers regarding the fear of a SARS-CoV-2 infection and the fear of COVID-19 symptoms had to be given on a visual analogue scale (VAS), which was 100 mm long and consisted of the two anchors “no fear” on the left and “maximum fear” on the right. The distance from the left anchor “no fear” was measured, and therefore higher scores indicate higher fear.

### 2.3. Statistical Analysis

The analysis was performed using IBM SPSS^®^ version 27. Descriptive statistics are presented as *n* and % for categorical variables and means ± standard deviations for continuous variables. In order to compare the reasons for and against vaccination in the two cohorts (before vs. after the official recommendation), chi-square tests were calculated. These tests were applied to pregnant and breastfeeding women independently. Bonferroni–Holm corrections were applied in order to account for Type 1 errors.

## 3. Results

### 3.1. Participant Characteristics

**Figure 1:**
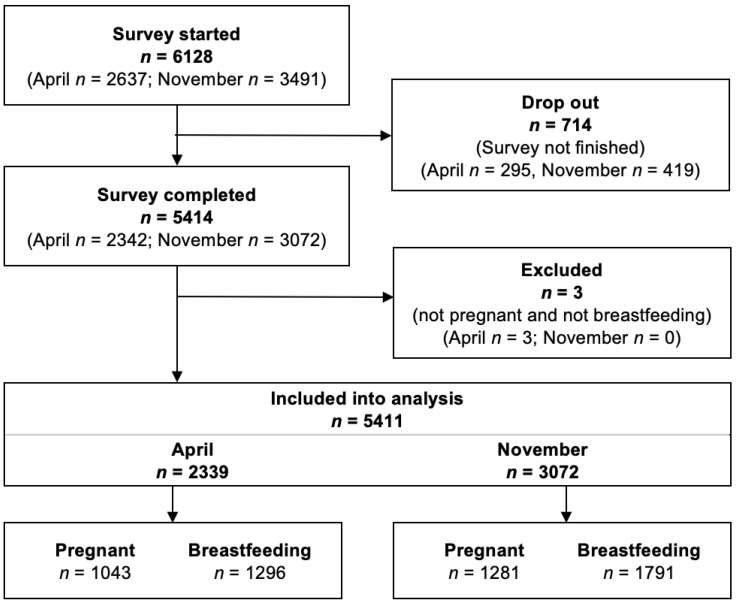
Flow chart of the study sample.

Overall, data from 5411 participants (42.9% pregnant; 57% breastfeeding) were analysed. A total of 2339 women (1043 pregnant and 1296 breastfeeding) completed the first survey, and 3072 (41.7% pregnant and 58.3% breastfeeding) completed the second. The study flow chart is presented in Figure 1. Regarding age, we compared the age distribution between the two cohorts (April and November) using chi-square tests, 12 which revealed no statistical significance for pregnant (*p* = 0.530) and breastfeeding women (*p* = 0.071). Further characteristics of the participants for the first and second surveys are shown in Table 1.

### 3.2. Vaccination Recommendation Awareness

The official recommendation for COVID-19 vaccination of pregnant and breastfeeding women was known to 95.0% of all participants—pregnant women at 95.1% and breastfeeding women at 94.9%.

**Figure 2:**
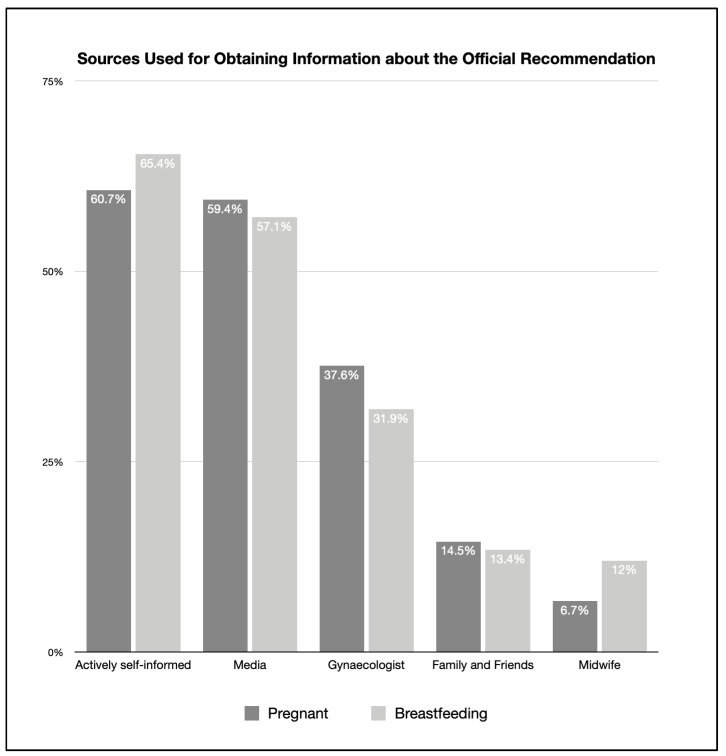
Sources used for obtaining information about the official recommendation (percentage of information sources used by participants after the release of the official recommendation; multiple answers possible).

Pregnant and breastfeeding women independently obtained information about the recommendation in 60.7% and 65.4%, respectively. Those who obtained the recommendation via a doctor were represented in 37.6% and in 31.9%, respectively. Those who were notified by a midwife accounted for 6.7% and 12.0%, respectively. Those who were informed through the media accounted for 59.4% and 57.1%, and those informed by friends or family accounted for 14.5% and 13.4%, respectively. These data are shown in Figure 2.

### 3.3. Pregnant Women’s Attitudes towards COVID-19 Vaccination

#### 3.3.1. Results from the Second Survey after the National Recommendation

Reasons for vaccination within the vaccinated pregnant group were more fear of the infection than of the side effects of vaccination (66.2%), intended protection of the baby and oneself (62.9%), work-related reasons (31.1%), societal reasons (26.2%), STIKO recommendation (15.7%), individual high SARS-CoV-2 exposure risk (15.3%), individual risk for severe COVID-19 (12.4%), and no specific reason (2.4%).

The reasons for rejection of the vaccination among pregnant women (*n* = 452) were answered as follows: due to the pregnancy itself (45.4%), general rejection of COVID-19 vaccination independent of pregnancy (25.0%), fundamental rejection of any vaccination in pregnancy (23.5%), and others (6.2%).

The influence of the official recommendation on their own vaccination decision was reported by 7.6% of the non-vaccinated pregnant women and 43.4% of the vaccinated pregnant women. Decision-making independent of the official recommendation was reported by 81.7% of the non-vaccinated pregnant women and by 51.5% of the vaccinated pregnant women. The answer “not sure” was stated by 8% of the non-vaccinated pregnant women and 3.3% of the vaccinated pregnant women.

The influence of gynaecologists’ advice on the women’s own vaccination status was reported by 17.0% of non-vaccinated pregnant women and by 42.5% of vaccinated pregnant women. In regard to the women’s gynaecologists’ advice, an independent decision was stated by 59.2% of the non-vaccinated pregnant women and by 37.8% of the vaccinated pregnant women. The issue of vaccination was not addressed by the gynaecologist in 18.5% of the unvaccinated and 15.4% of the vaccinated pregnant women. The answer “not sure” was stated by 5.3% of the non-vaccinated pregnant women and 4.3% of the vaccinated pregnant women.

#### 3.3.2. Comparison before and after the Official Recommendation

**Figure 3:**
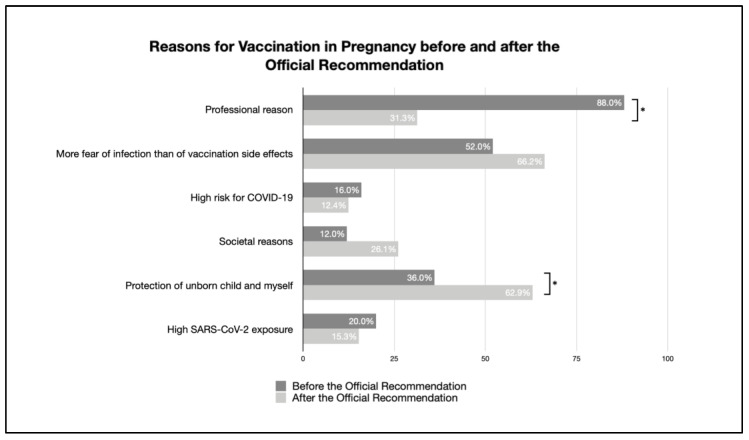
Reasons for vaccination in pregnancy before and after the official recommendation (percentage of reasons given by participants for being vaccinated; multiple answers possible; * indicates a significant difference).

**Figure 4:**
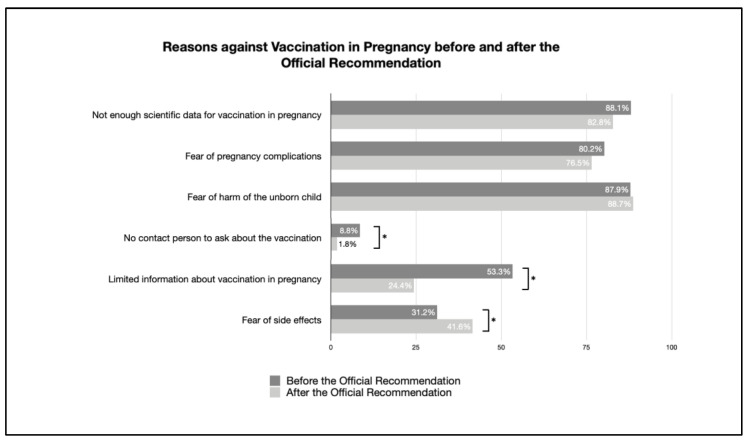
Reasons against vaccination in pregnancy (percentage of reasons given by participants against being vaccinated; multiple answers possible; * indicates a significant difference).

The reasons for and against vaccination—when comparing both surveys before and after the publication of the official recommendation—are given in Table 2. Furthermore, changes are clearly visible in Figure 3 and Figure 4.

The following reasons for vaccination during pregnancy changed when comparing the data from before and after the official recommendation. Specifically, the reason to protect oneself and the unborn child (36.0–62.9%) represented a significant change, and the increased fear of infection rather than possible side effects (52.0–66.2%) raised concerns. On the other hand, the motivation to be vaccinated for professional reasons decreased (88.9–31.3%).

Among the reasons for not vaccinating, all aspects have decreased, or at least are not significantly different, except for the significant increase in the fear of side effects.

### 3.4. Breastfeeding Women’s Attitudes towards COVID-19 Vaccination

#### 3.4.1. Results from the Second Survey after the National Recommendation

The reasons for vaccination among the vaccinated breastfeeding women (*n* = 1338) were: more fear of the infection than of the side effects of vaccination (67.0%), intended protection of the newborn and self-protection (89.2%), professional reasons (14.7%), societal reasons (37.6%), STIKO recommendation (17.8%), individual high SARS-CoV-2 exposure risk (7.1%), individual risk for severe COVID-19 (7.5%), and no specific reason (1.3%).

Reasons mentioned in the context of not wanting to become vaccinated among breastfeeding women (*n* = 254) were the lactation itself (29.9%), the fundamental rejection of COVID-19 vaccination independent of lactation (39.4%), the fundamental rejection of any vaccination during lactation (25.2%), and other reasons (5.5%).

The self-reported influence of the official recommendation on the decision to become vaccinated or not in the lactation period was stated by 15.8% of the not vaccinated breastfeeding women and by 46.3% of the vaccinated group.

In addition, the official recommendation of an independent decision was reported by 68.4% of the non-vaccinated breastfeeding women and by 48.2% of the vaccinated. The answer “not sure” was advised by 10.2% of the non-vaccinated breastfeeding women and 3.7% of the vaccinated breastfeeding women.

The self-reported influence of gynaecologists’ advice on their own vaccination status was reported by 11.7% of the non-vaccinated breastfeeding women and by 39.6% of the vaccinated breastfeeding women. The reason for the gynaecologists’ advice and independent decision was stated by 48.6% of the non-vaccinated breastfeeding women and by 33.3% of the vaccinated breastfeeding women. The answer “not sure” was advised by 4.6% of the non-vaccinated breastfeeding women and 2.4% of the vaccinated breastfeeding women.

Vaccination was not specifically mentioned by the gynaecologist in 35.1% of the unvaccinated and in 24.8% of the vaccinated breastfeeding women.

#### 3.4.2. Comparison before and after Official Recommendation

**Figure 5:**
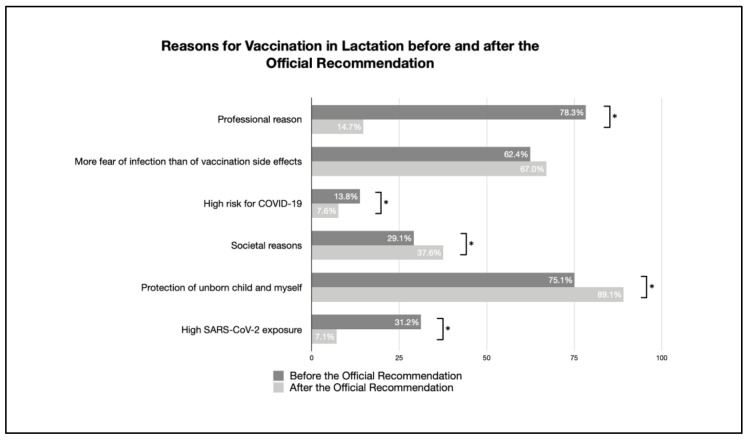
Reasons for vaccination in lactation before and after the official recommendation (percentage of reasons given by participants for being vaccinated; multiple answers possible; * indicates a significant difference).

**Figure 6:**
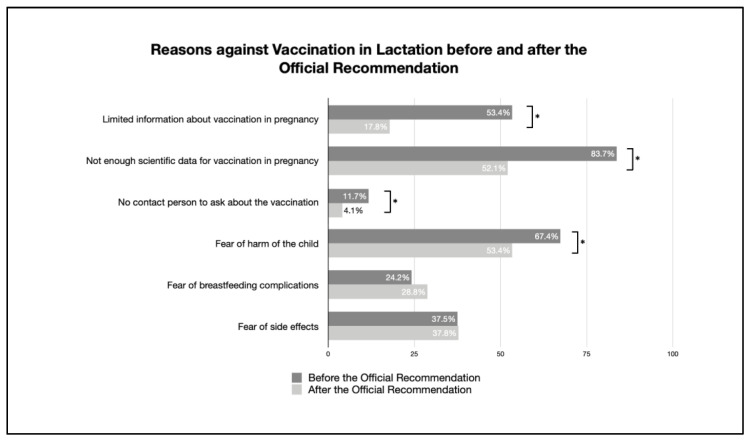
Reasons against vaccination in lactation before and after the official recommendation (percentage of reasons given by participants against being vaccinated; multiple answers possible; * indicates a significant difference).

The reasons for and against vaccination, both before and after publication of the official recommendation regarding the vaccination of breastfeeding women, are given in Table 3, and changes are visibly discernible in Figure 5 and Figure 6.

The reasons for vaccination during lactation significantly increased, i.e., to protect oneself and the unborn child (75.1–89.1%) as well as for societal reasons (29.1–37.6%). The reasons for being vaccinated for work-related reasons (78.3–14.7%), the reasons for a higher exposure to SARS-CoV-2 (31.2–7.1%), and the higher risk of severe COVID-19 disease symptoms (13.8–7.6%) decreased.

Moreover, there was no difference in the perception of being more afraid of infection than of possible side effects.

Most reasons for not being vaccinated were significantly reduced: limited information about vaccination during lactation, not enough scientific data for vaccination, no contact person to ask about the vaccination, and fear of harm to the child. There was no significant difference in the fear of side effects and complications during breastfeeding.

### 3.5. Identification of General COVID-19 Vaccination Opponents

**Figure 7:**
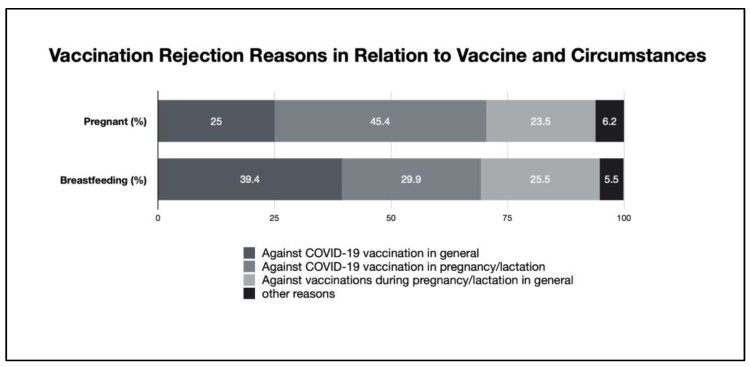
Vaccination rejection in relation to vaccine and circumstances.

In order to identify and differentiate general COVID-19 vaccine opponents from those who are solely against COVID-19 vaccination in pregnancy/lactation or who are against vaccination in general in obstetric circumstances, we questioned these matters as distinct within the unvaccinated group. The results can be broken down as follows: (1) against COVID-19 vaccination in general and would also not be vaccinated if not pregnant/breastfeeding were 25% of the pregnant (*n* = 425) and 39.4% of the breastfeeding (*n* = 254) women; (2) against COVID-19 vaccination in pregnancy/lactation were 45.4% and 29.9%; and (3) against vaccinations during pregnancy/lactation in general were 23.5% and 25.5%, respectively. Other reasons were given by 6.2% and 5.5%, respectively. These data are shown in Figure 7.

## 4. Discussion

This study aimed to investigate the vaccination prevalence, the reasons for and against vaccination, and their changes in relation to the official national recommendations among pregnant and breastfeeding women. In addition, we assessed the level of awareness of this recommendation and the source from which this information was obtained.

### 4.1. Vaccination Prevalence

In November 2021, after the official recommendation, the vaccination uptake among pregnant women in Germany was 58.7%, which was below the overall German average of 71% [13]. Among breastfeeding women, however, the vaccination rate was higher at 74.7%.

At that time, the vaccination rates were comparable to those in other industrialised countries worldwide. Germany ranked in the average range (France 77%, UK 75%, US 68%, Israel 66%, and Switzerland 66%) [13].

### 4.2. Reasons for and against Vaccination

Regarding the reasons for vaccination, we were able to show in the group of pregnant women that the relative amount of those vaccinated for work-related reasons has decreased over time. Due to access restrictions at the beginning of the vaccination programme in early 2021 for job-exposed and vulnerable population groups, this development can be satisfactorily explained. In relation to the established “3 C-model” on vaccination hesitancy (including confidence, complacency, and convenience) of the SAGE working group, the point of convenience was thus improved with the broad accessibility to the vaccine [14]. Regarding complacency, a higher perceived risk of COVID-19 was a key motivator to become vaccinated [15]. Accordingly, the rate of pregnant and lactating women who advocated vaccination in order to protect themselves and their unborn children increased. The other major factor for COVID-19 vaccination in our study was the outweighing of the fear of infection more so than the side effects of the vaccination, which was the case in both pregnant and especially breastfeeding women. These findings are in line with the results of other studies [16,17,18]. The main reasons against vaccination were fear of complications during pregnancy/breastfeeding and fear of harm to the unborn/newborn, which is also consistent with the results of other studies [3,19].

A further identified motivator was trust and confidence (“3 C-model”) in the health care system [14,15,19], which is represented in our study by the official STIKO recommendation. After publishing these data, the proportion of those indicating too little information as a reason for not being vaccinated more than halved among pregnant women (53.5% vs. 24.4%). Moreover, only one-third remained among breastfeeding women (53.4% vs. 17.8%).

Our findings are in line with the results of a survey with 18,874 participants, which showed a high level of trust in the German Robert Koch Institute, to which the STIKO belongs [20]. This was proportionately higher among women than men. However, trust decreased during the pandemic. This underlines the importance of continuing educational measures.

### 4.3. Knowledge Transfer and Dissemination of the Official Recommendation

The information regarding the official recommendation was obtained mainly autonomously or through media exposure; this underlines the rate of awareness of the vaccination topic by the obstetric target groups, i.e., easy access to the current information and media exposure.

Only in less than one-third of the respondents did the gynaecologist or, even more rarely, the midwife provide the information. Clearly, there is more potential to address the targeted dissemination of scientific data.

Furthermore, this would be of particular importance insofar as, fortunately, the information was well disseminated with a tremendous level of awareness. However, the perception that there is not enough scientific data was not so different before and after the official recommendation, as stated by the pregnant women. Even though there was a steady increase in robust evidence on the safety and efficacy of the COVID-19 vaccination published during this period [21,22,23]. This indifference was not detected in the breastfeeding group, as the rate of those who gave not enough scientific data as a reason for not being vaccinated decreased significantly.

This raises the question of whether the presentation of the content of scientific information is appropriate for the target group in terms of conveying the key messages of evidence. A modification of the content presentation may be worth considering. There is evidence that the combination of different media types increases vaccination uptake [24]. A complementary use of media based on the usual website presentation with additional content provision via social media offers promising approaches. Regarding the use of social media as a communication platform, future research on trust in institutions should investigate whether the target group with low trust in institutions can be reached on relevant media, such as messenger services and social media platforms. However, a detailed investigation is urgently recommended beforehand in order to exclude opposing and adverse effects [25].

### 4.4. The Role of Health Care Professionals

The fact that the vaccination uptake among pregnant women is below the national average may be explained due to the fact that only a small proportion of gynaecologists have addressed the recommendations and the COVID-19 vaccination. As we previously identified, when asked who was the preferred contact for questions about the COVID-19 vaccination, 88.1% of pregnant women named their gynaecologist [12].

Moreover, evidence already exists pre-pandemic that a physician’s recommendation to become vaccinated is the most important factor in maternal decision-making [26,27].

The dissemination of up-to-date scientific data to the affected groups is ineffective and can therefore cause lasting (though avoidable) harm for both the mother-to-be and her offspring. It is questionable whether the key health-care professional interlocutors (gynaecologists, midwives, etc.) are constantly up-to-date with the rapid and dynamic development of evidence. Additionally, it is conceivable that the high frequency of publications and new data on the vaccination led to uncertainty on the part of the gynaecologists in the counselling situation, thereby resulting in a negative synergy with regard to the vaccination uptake.

Hence, there is a need, but also an opportunity, to optimise the provision of information to healthcare professionals themselves, as they act as important multipliers. Chervenak et al. noted, regarding counselling, that rather than highlighting the risk of the disease itself, focusing on the protective role and safety of the vaccine in pregnant and lactating women would prove more beneficial [28].

### 4.5. Vaccination Opponents

Furthermore, we identified the proportion of COVID-19 vaccination opponents. This differentiation among the unvaccinated has only rarely been recorded thus far and, to our knowledge, has not yet been evidenced in an obstetric sample. At 6.9% of the total collective, this proportion is similar to the 8% described by Motta et al. in their survey (*n* = 1001; USA, general population) [29]. In our study, the proportion of vaccination opponents among the unvaccinated is almost one third and represents a major challenge for the health system. This is a further aspect of the confidence “C” (3 C-model, see above), which is defined, inter alia, as trust in the system that delivers vaccines, including the reliability and competence of the health services and health professionals [14].

As Motta et al. showed, the rejection of vaccination is part of a self-ideology that is difficult to overcome due to its complexity. With the ever-growing acceptance of vaccination in the population, the proportion of “anti-vaxxers” will also increase, thereby making it more difficult to overcome the hesitancy and rejection of these people towards vaccination if they perceive this label as an important part of their self-image.

### 4.6. Clinical Implications

A target group-oriented presentation of scientific evidence should be promoted to a greater extent. This study implies that understandable language and presentation via different media types, where appropriate, are strong ways to communicate the importance of vaccination to pregnant women and breastfeeding mothers. These are findings that are likely to be transferable in an international context.

In particular, we were able to demonstrate through the current work that medical professionals, as confidants and multipliers, need to be educated continuously in the context of rapidly evolving evidence. This represents a key interface for convincing undecided people of the benefits of vaccination in a clear and evidence-based way. In addition, they need to be encouraged and sensitised to actively address and recommend vaccination as the most important protective measure against COVID-19.

### 4.7. Strengths and Limitations

To the best of our knowledge, this is the largest study so far that has investigated the dynamics regarding the reasons for and against COVID-19 vaccination in respect of pregnant and breastfeeding women. The large sample size speaks for the resilience of the survey results for pregnant and breastfeeding women in Germany. However, an accurate assessment regarding generalisability is not possible due to open recruitment issues. Although we acknowledge that we have not assessed the socio-demographic background of the sample in depth, we could show that the age distribution did not significantly differ between the two surveys, and the mean ages are comparable to the average age of pregnant women in Germany in 2021 (which was 30.8 years; 30.5 years at the first pregnancy, 32.4 years at the second, and 33.5 years at the third) [30]. However, in the breastfeeding sample, there was a significant trend regarding the age distribution. Although this is beyond the scope of the present study, it would be highly interesting to investigate in future studies whether age is related to vaccine preferences.

With our study, we identify which potentials can still be tapped in order to implement strategic measures to improve vaccination adherence, with special consideration of vulnerable population groups such as pregnant and breastfeeding women.

Due to the design of an open, online-based survey, methodological selection bias must be considered when interpreting the results, as well as the fact that a non-validated questionnaire was used. Specifically, possible bias is based on both access to social media during recruitment and online-based survey responses as well as the participant’s intention to participate, depending on their advocacy for or opposition to vaccination in general, or in the case of pregnancy and breastfeeding in particular. Hence, following the above argumentation, we cannot exclude the probability of a sample bias with complete certainty. Due to the recruitment methodology, web-based access to the survey, and the intention of participation, limitations in the comparability of both survey parts have to be taken into account in the interpretation.

As a further limitation, we here investigate the influence of the official recommendation on vaccine preferences in two different samples. We acknowledge that a within-subject design would have been preferable in order to minimise selection biases and exclude differences in baseline characteristics that may influence the results. However, this was not possible as an anonymous online questionnaire was administered in order to get the questionnaire online quickly during a rapidly changing pandemic and in order to gain a large sample size.

As a limitation, results are not entirely transferable to other countries, as access to vaccination and official national recommendations were given at different times during the pandemic. Furthermore, in Germany, ethnic origin is only of minor importance, as this only applies to a very small part of the population.

Given that not only the COVID-19 pandemic but also significant other influences affect humanity in the 21st century [1], certain behavioural patterns and decision-making effects influenced by anxiety and stress cannot be attributed to the pandemic alone.

### 4.8. Research Implications

Based on our analysis, further issues have arisen and are worth exploring in order to improve effective care, which is to say that the following should be investigated: the influence of social media on the willingness to vaccinate and the resulting possibility of directly presenting scientifically prepared data to corresponding target groups.

The question of how healthcare professionals can be kept up-to-date more easily and effectively in the specific situation of rapidly growing data, such as during a pandemic, is an important one to properly consider and answer.

Various strategies to counter vaccine scepticism in counselling have been established—whether professionals can be trained effectively and whether this offer would be taken up alongside the daily routine would be a step towards containing the pandemic.

## 5. Conclusions

A high level of awareness is present regarding official national recommendations for COVID-19 vaccination in pregnant and breastfeeding women. This information was obtained mostly autonomously. Furthermore, this is reflected in the higher vaccination uptake among pregnant women in the context of an international comparison, as well as among breastfeeding women above the overall German population average of vaccination uptake.

Pre- and post-partum fear of infection outweighed the fear of vaccination side effects. In addition, the protection of the offspring and oneself were important reasons for vaccination, which increased during the pandemic.

The perception of scientific evidence through official recommendations can potentially be improved through targeted, tailored education strategies.

Lastly, healthcare professionals, in their key role as multiplicators of evidence-based information and main confidants in this context, should be more engaged.

## Figures and Tables

**Table 1:** Characteristics of pregnant and breastfeeding women.

	Before Official Recommendation (*n* = 2339)		After Official Recommendation (*n* = 3072)	
	Pregnant Women	Breastfeeding Women	Pregnant Women	Breastfeeding Women
*n*	1043	1296	1281	1791
Age (years)	31.8 ± 4.3	32.4 ± 4.4	30.8 ± 4.0 (20–46)	31.7 ± 4.3
Age distribution (*n*)				
≤24 y	48	47	58	65
25–39 y	959	1172	1189	1652
≥40 y	36	77	34	74
Medical condition with higher risk for COVID-19	210 (20.1%)	262 (20.2%)	198 (15.5%)	207 (11.5%)
SARS-CoV-2 positive previously	44 (4.2%)	45 (3.7%)	96 (7.5%)	106 (5.9%)
COVID-19 symptoms No symptoms Mild symptoms Severe symptoms Hospitalised	6 (13.6%) 30 (68.2%) 8 (18.2%) -	5 (11.1%) 35 (77.8%) 5 (11.1%) -	8 (8.3%) 61 (63.5%) 25 (26%) 2 (2.1%)	4 (3.8%) 80 (75.5%) 20 (18.9%) 2 (1.9%)
Fear of COVID-19 symptoms (VAS *; mm)	57.12 ± 27.09	55.14 ± 26.60	54.1 ± 27.0	54.2 ± 26.1
Fear of SARS-CoV-2 infection (VAS *; mm)	51.18 ± 25.55	48.06 ± 24.53	52.1 ± 25.0	50.6 ± 24.5
Gestational age (weeks)	24.7 ± 9.1	-	26.2 ± 9.5 (4–42)	-
1. Trimester	12% (116/966)		8.4% (108/1279)	
2. Trimester	34% (330/966)		34.5% (442/1279)	
3. Trimester	53.8% (520/966)		57.0% (729/1279)	
First pregnancy	494 (47.4%)	-	804 (63%)	-
High-risk pregnancy	418 (40.1%)	-	401 (31.1%)	-
COVID-19 vaccination				
no yes	1018 (97.6%) 25 (2.4%) 1 dose 13 (52%) 2 doses 12 (48%)	1108 (85.5%) 188 (14.5%) During pregnancy 2 (1.1%) Post-partum 186 (98.9%)	529 (41.3%) 752 (58.7%) 1 dose 86 (11.4%) 2 doses 660 (87.8%) 3 doses 6 (0.8%)	452 (25.3%) 1339 (74.8%) During pregnancy 453 (33.8%) Post-partum 1116 (83.3%)

* VAS: Visual Analogue Scale, mm (0–100).

**Table 2:** Comparison of reasons for and against COVID-19 vaccination in pregnancy in relation to the publication of the STIKO recommendation.

	Before Official Recommendation *n* = 2339	After Official Recommendation *n* = 3072	*p*-Values	Effect Size ϕ
Pregnant women’s reason for vaccination				
Professional reason	88.0% (22/25)	31.3% (235/752)	<0.001	0.213
More fear of infection than of vaccination side effects	52.0% (13/25)	66.2% (498/752)	0.052	.
High risk for COVID-19	16.0% (4/25)	12.4% (93/752)	0.589	
Societal reasons	12.0% (3/25)	26.1% (196/752)	0.113	
Protection of the unborn child and myself	36.0% (9/25)	62.9% (473/752)	0.003	0.098
High SARS-CoV-2 exposure	20.0% (5/25)	15.3% (115/752)	0.522	
Official STIKO recommendation	n/a	15.7% (118/752)	-	
Pregnant women’s reason against vaccination				
Limited information about vaccination during pregnancy	53.5% (229/430)	24.4% (54/221)	0.001	0.275
Not enough scientific data for vaccination during pregnancy	88.1% (379/430)	82.8% (183/221)	0.062	
No contact person to ask about the vaccination	8.8% (38/430)	1.8% (4/221)	0.006	0.135
Fear of harm to the unborn child	87.9% (378/430)	88.7% (196/221)	0.770	
Fear of pregnancy complications	80.2% (345/430)	76.5% (169/221)	0.265	
Fear of side effects	31.2% (134/430)	41.6% (92/221)	0.032	0.104

STIKO: Standing Vaccination Committee at the Robert Koch Institute, Germany.

**Table 3:** Comparison of reasons for and against COVID-19 vaccination in breastfeeding women in relation to the publication of the STIKO recommendation.

	Before Official Recommendation	After Official Recommendation	*p*-Values	Effect Size ϕ
Breastfeeding women’s reason for vaccination				
Professional reason	78.3% (148/189)	14.7% (197/1339)	<0.001	0.501
More fear of infection than of vaccination side effects	62.4% (118/189)	67.0% (897/1339)	0.241	
High risk for COVID-19	13.8% (26/189)	7.6% (102/1339)	0.012	0.073
Societal reasons	29.1% (55/189)	37.6% (502/1339)	0.048	0.058
Protection of my child and myself	75.1% (142/189)	89.1% (1193/1339)	<0.001	0.138
High SARS-CoV-2 exposure	31.2% (59/189)	7.1% (95/1339)	<0.001	0.264
Official STIKO recommendation	n/a	46.3% (619/1338)	-	
Breastfeeding women’s reason against vaccination				
Limited information about vaccination during lactation	53.4% (141/264)	17.8% (26/146)	<0.001	0.347
Not enough scientific data for vaccination during lactation	83.7% (221/264)	52.1% (76/146)	<0.001	0.339
No contact person to ask about the vaccination	11.7% (31/264)	4.1% (6/146)	0.03	0.128
Fear of harm to the child	67.4% (178/264)	53.4% (78/146)	0.02	0.138
Fear of breastfeeding complications	24.2% (64/264)	28.8% (42/146)	0.316	
Fear of side effects	37.5% (99/264)	37.7% (55/146)	0.973	

## Data Availability

The datasets generated and/or analysed during the current study are not publicly available due to reasons of data protection but are available from the corresponding author on reasonable request.
